# Experimental method for 3D reconstruction of Odonata wings (methodology and dataset)

**DOI:** 10.1371/journal.pone.0232193

**Published:** 2020-04-29

**Authors:** Nasim Chitsaz, Romeo Marian, Javaan Chahl

**Affiliations:** 1 School of Engineering, University of South Australia, Adelaide, SA, Australia; 2 Joint and Operations Analysis Division, Defence Science and Technology Group, Melbourne VIC, Australia; University of New South Wales, AUSTRALIA

## Abstract

Insect wings are highly evolved structures with aerodynamic and structural properties that are not fully understood or systematically modeled. Most species in the insect order Odonata have permanently deployed high aspect ratio wings. Odonata have been documented to exhibit extraordinary flight performance and a wide range of interesting flight behaviors that rely on agility and efficiency. The characteristic three-dimensional corrugated structures of these wings have been observed and modeled for a small number of species, with studies showing that corrugations can provide significant aerodynamic and structural advantages. Comprehensive museum collections are the most practical source of Odonata wing, despite the risk of adverse effects caused by dehydration and preservation of specimens. Museum specimens are not to be handled or damaged and are best left undisturbed in their display enclosures. We have undertaken a systematic process of scanning, modeling, and post-processing the wings of over 80 Odonata species using a novel and accurate method and apparatus we developed for this purpose. The method allows the samples to stay inside their glass cases if necessary and is non-destructive. The measurements taken have been validated against micro-computed tomography scanning and against similar-sized objects with measured dimensions. The resulting publicly available dataset will allow aeronautical analysis of Odonata aerodynamics and structures, the study of the evolution of functional structures, and research into insect ecology. The technique is useable for other orders of insects and other fragile samples.

## Introduction

**Fig 1:**
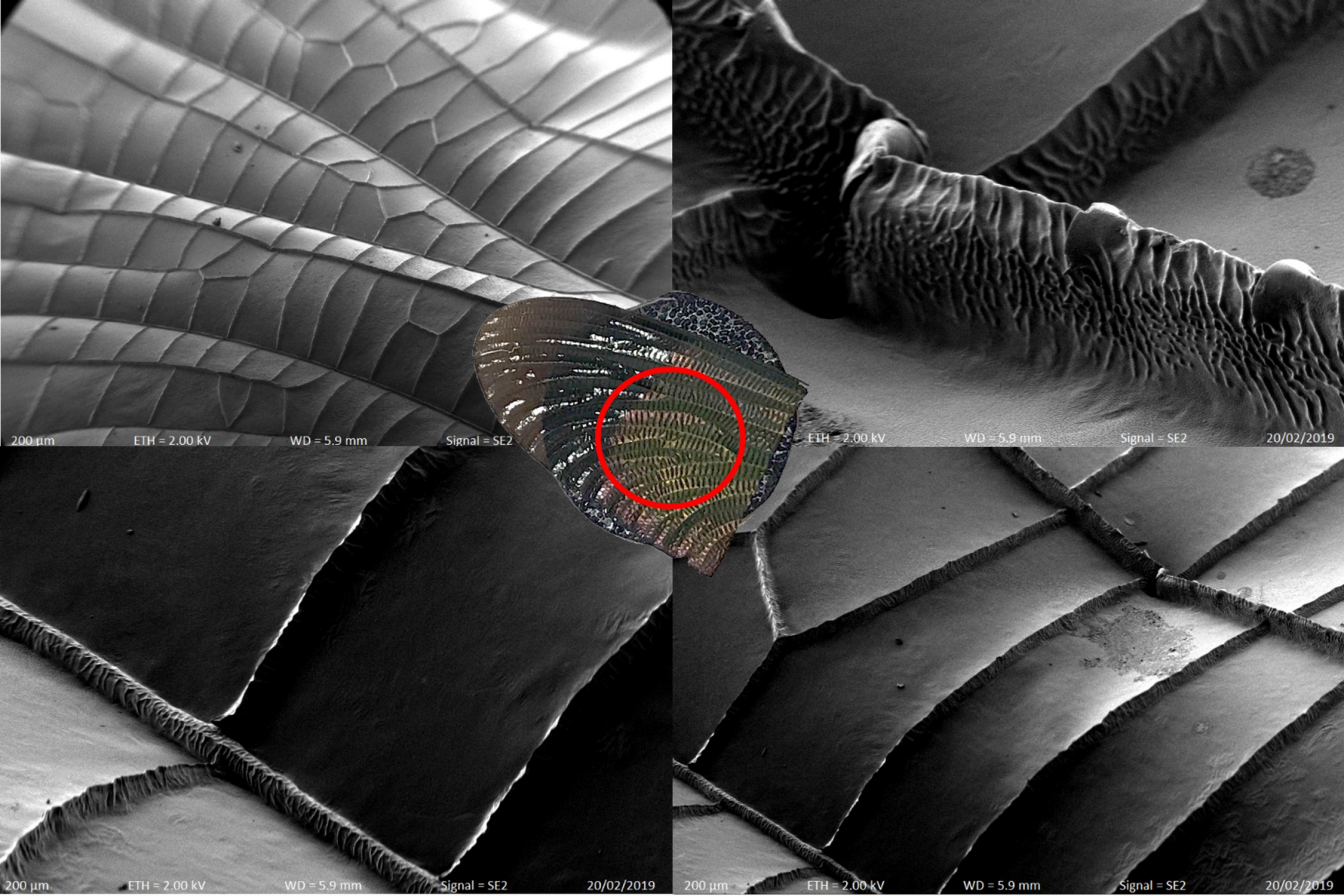
SEM image of *Neurobasis Daviesi* wing.

Insect and bird wings are an evolved, functional structure that have been subject to an unusual number of evolutionary pressures, including aerodynamics [1]. In some regards, dragonfly wings, with their permanently deployed position, may be among the purest examples of animal wings evolved for aerodynamics. Detailed analysis of the design of insect wings may provide new insights into how insect wings have been adapted towards improved aerodynamics by evolution [2]. Proto-Odonata were among the first winged insects to evolve, over 300 million years ago [3, 4]. Many modern Odonata fly fast, are highly maneuverable [5], and can fly over long ranges [6]. These characteristics, associated with efficient and effective flight, compare favorably with other orders of the insect [7]. From the perspective of an existence proof, large Odonata wings are an attractive candidate model for micro air vehicles (MAVs) [8, 9]. The wings of Odonata are corrugated in the millimeter scale, rather than being smooth [10, 11] and are likely to produce lift and drag in complex ways, while structurally deforming under aerodynamic loads. The structure of Odonata wings has been shown to play an important role during flight because the deformation of the wings changes their aerodynamics [12]. An accurate model of the structure is necessary to allow the use of computational techniques to explore the interactions between aerodynamic forces and the structure of the wing [13–15]. Fig 1 shows the corrugation and structure of the *Neurobasis Daviesi* wing surface captured with a scanning electron microscopy (SEM), which was conducted at the University of South Australia. Structure is present at the millimeter through to micron scale. For aerodynamic and mechanical purposes, structures of the order of tens of microns are likely to be relevant.

**Fig 2:**
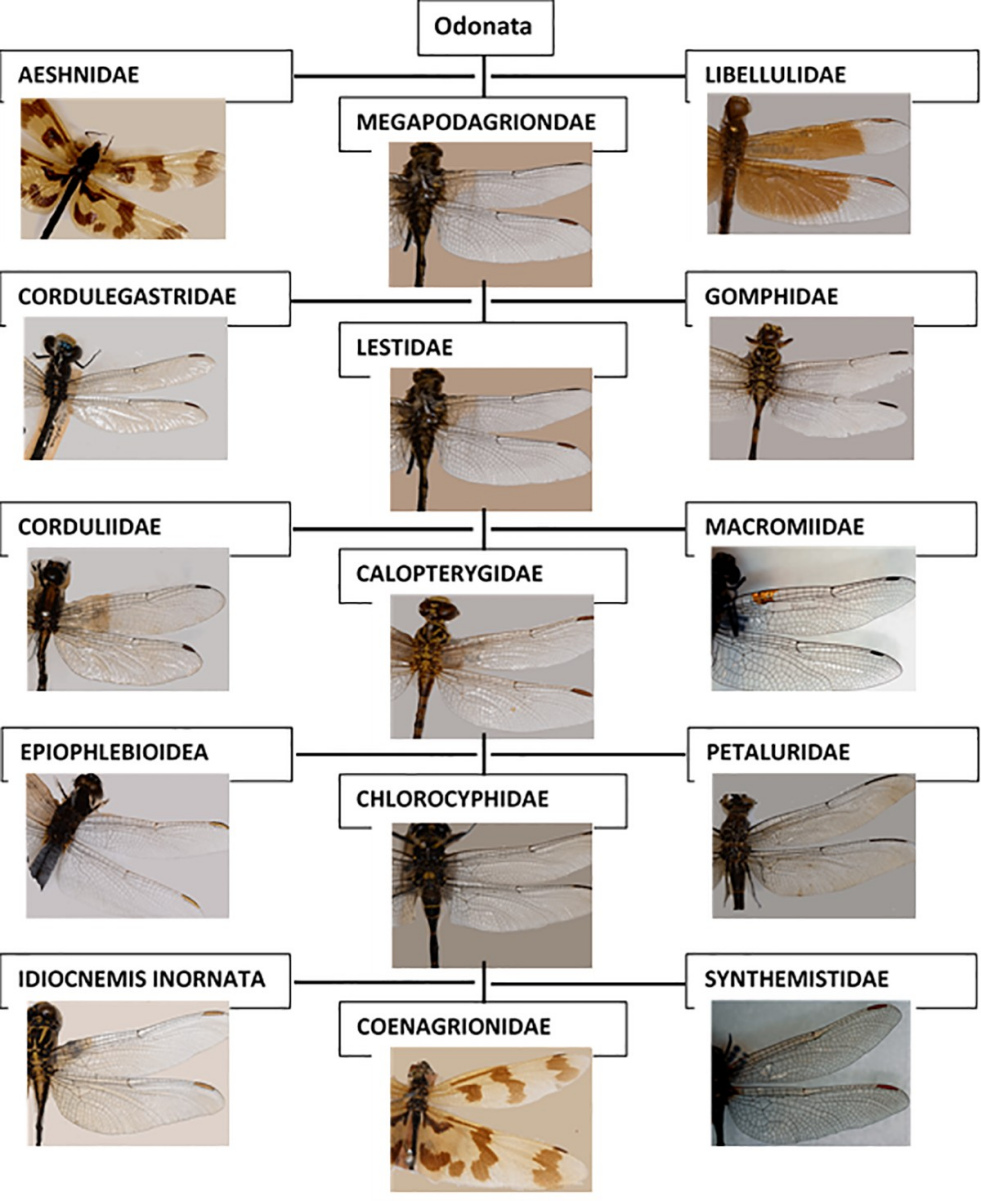
Odonata classification and diversity of the dataset.

Odonata are phylogenetically classified into three suborders: *Epiprocta* and *Zygoptera* [4, 16]. Each of these suborders is further divided into superfamily and then family. Fig 2 shows the order of Odonata and their subcategories.

Seeking Odonata in natural environments for scanning would require significant resources in time, travel, and personnel. Many Odonata require undisturbed and difficult to access environments, and many species are scarce with populations that are threatened by human activity. For these reasons, it is impractical and improper to gather a large sample of Odonata species for one purpose. Comprehensive collections of specimens exist throughout the world in museums, potentially allowing the wings of all known species to be modeled.

Disturbances, let alone destructive tests and energy emitting digitization technologies, should not be suffered by dragonfly wings in the world’s collections. Past studies have tended towards destructive measurement technologies. For instance, in one study, an analysis of 26 individuals of the suborder of *Anisoptera* was performed in which the fore and hind wings were cut off and put under a scanner [2].

Similarly, Blanke conducted a photo scan test in order to analyze the variation of wing vein configuration by cutting off 198 wings of dragonflies and placing the wings under glass [17]. The dataset included raw data but lacked a 3D model [17]. Different methods have been applied to make a 3D model, such as photogrammetry aided by a laser spot [18–20]. 3D models have also been generated from a 3D laser scanner, as evidenced in various areas of research, such as an analysis of the texture of water surfaces [21, 22]. However, using a 3D laser scanner can be a slow and expensive procedure, and it injects substantial energy into the sample. We have demonstrated that even low power lasers used in these applications can be destructive to delicate wing membranes, which we will show in the discussion part of this paper.

Photogrammetry, on the other hand, is a fast and non-destructive process that emits no coherent energy and can measure through glass, which we have implemented using a normal digital camera with a computer-controlled mechanical slide [23]. Photogrammetry is an established method for fusing multiple two-dimensional images into a three-dimensional height map [24, 25]. An advantage of Photogrammetry is that the original images can be preserved for a later time when improved algorithms may become available, increasing the value of the approach for archival purposes.

## Materials and methods

No permits were required for the described study, which complied with all relevant regulations.

Wing parameters were measured for 80 individuals of eight different families of Odonata in the collection of the South Australian (SA) Museum, Adelaide, Australia. All species were adult and collected from Australia and other contries as specified in S1 Table. Odonata wings are formed on emergence and only degrade after that point. Good museum specimens will thus tend to be relatively recently emerged. Detailed information about the specimen suborder, family, and species are presented in S1 Table according to the museum database. The purpose of this study was to extract models of both the forewing (FW) and hindwing (HW), that each play significant roles in the aerodynamic performance of Odonata [26, 27]. One side of each wing pair was chosen for photogrammetric analysis and incorporation into the database. Through photogrammetry, the precise positions of surface points can be recovered using the apparent displacement of features in the photographs when individual features are visible in two or more photographs [28–30]. Optics, feature detection, and projective geometry are amongst the main principals of photogrammetry [30, 31].

**Fig 3:**
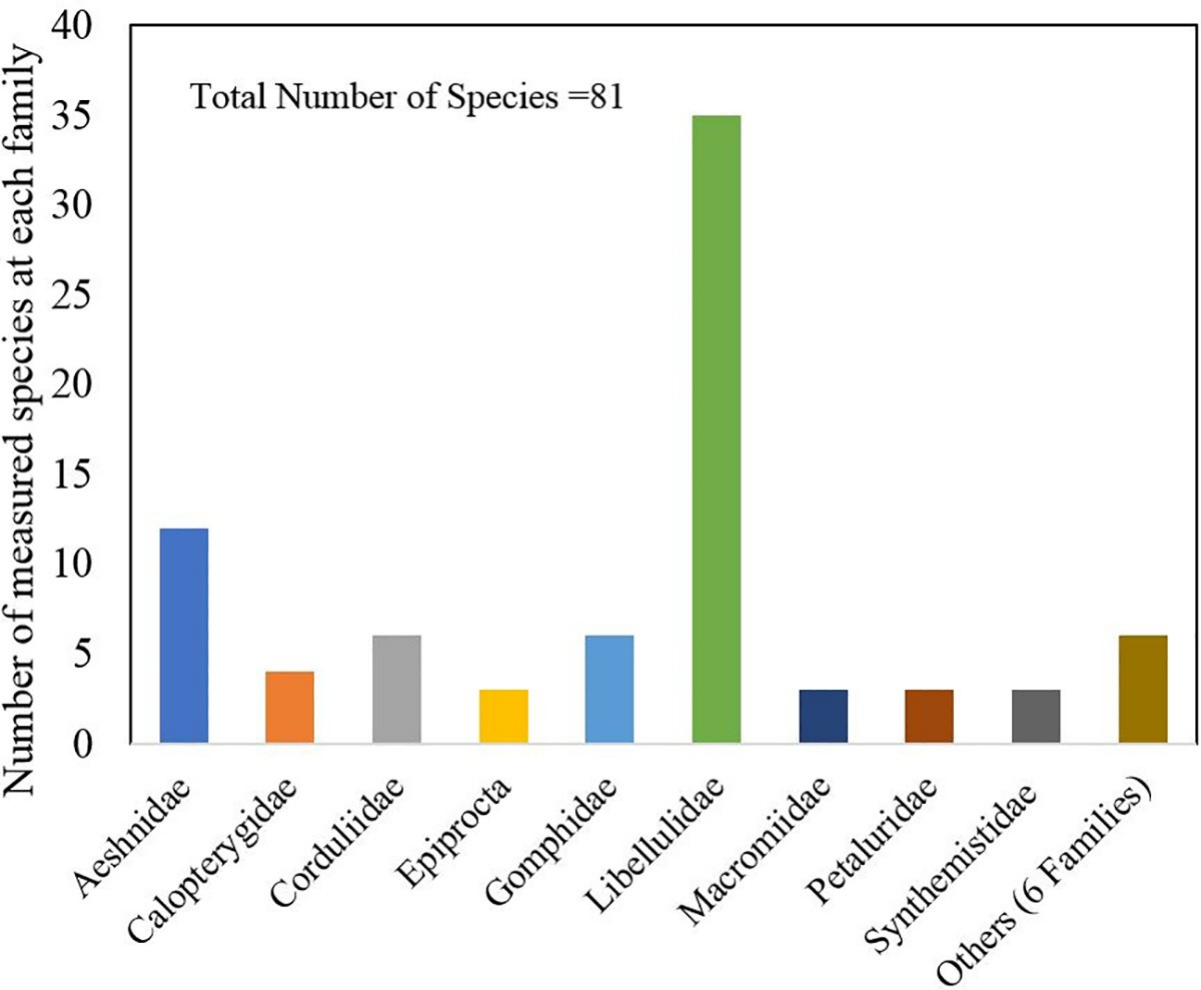
Total number of species and its population.

Preservation of the Odonata samples was the priority in this research to comply with the standards of the museum. We collected as many wing image-samples as possible. The method could not disturb the samples by any means. Handling was restricted to opening the sliding drawers holding the specimen cases and viewing through the glass, not removing or directly handling the samples. The photos taken were then “stitched” together using software to form a 3D image. With this technique, all texture, structure, and color on the top side of the wing were captured, and the original photographs were preserved in the database for future re-analysis. The representation of families within the database is shown in Fig 3. Nearly 50% of the dataset is composed to *Libellulidae* and *Aeshnidae* dragonfly families, the species which tend to be larger and show significant variation in size and appearance [32], which is correlated with their display value in museums.

### Experimental set-up and photogrammetric model

The equipment used to take photographs of the various wings, consisted of a Nikon D610 Digital single-lens reflex (DSLR) camera and macro lens (AF-S macro Nikkor 105 mm), LED lens-mounted soft light source (not a flash), professional tripod and a custom, computer-controlled, precision linear motion stage which allowed multiple images to be taken along a single axis, similar to a camera slider. A DSLR camera was chosen to allow repeatable control of exposure values. The metadata relating to exposure and aperture settings were automatically preserved in the images by the camera. Details of the procedure for data acquisition and measurement configurations are given in Table 1.

**Table 1:** Photographic acquisition data and measurement configurations.

Aperture value	f/10
**Overlap on each photo**	70–90%
**Camera**	Nikon D610
**Lens**	AF-S macro Nikkor 105 mm
**Distance to object**	22 cm
**Focal length**	105 m
**Lens angle**	0° degree

**Fig 4:**
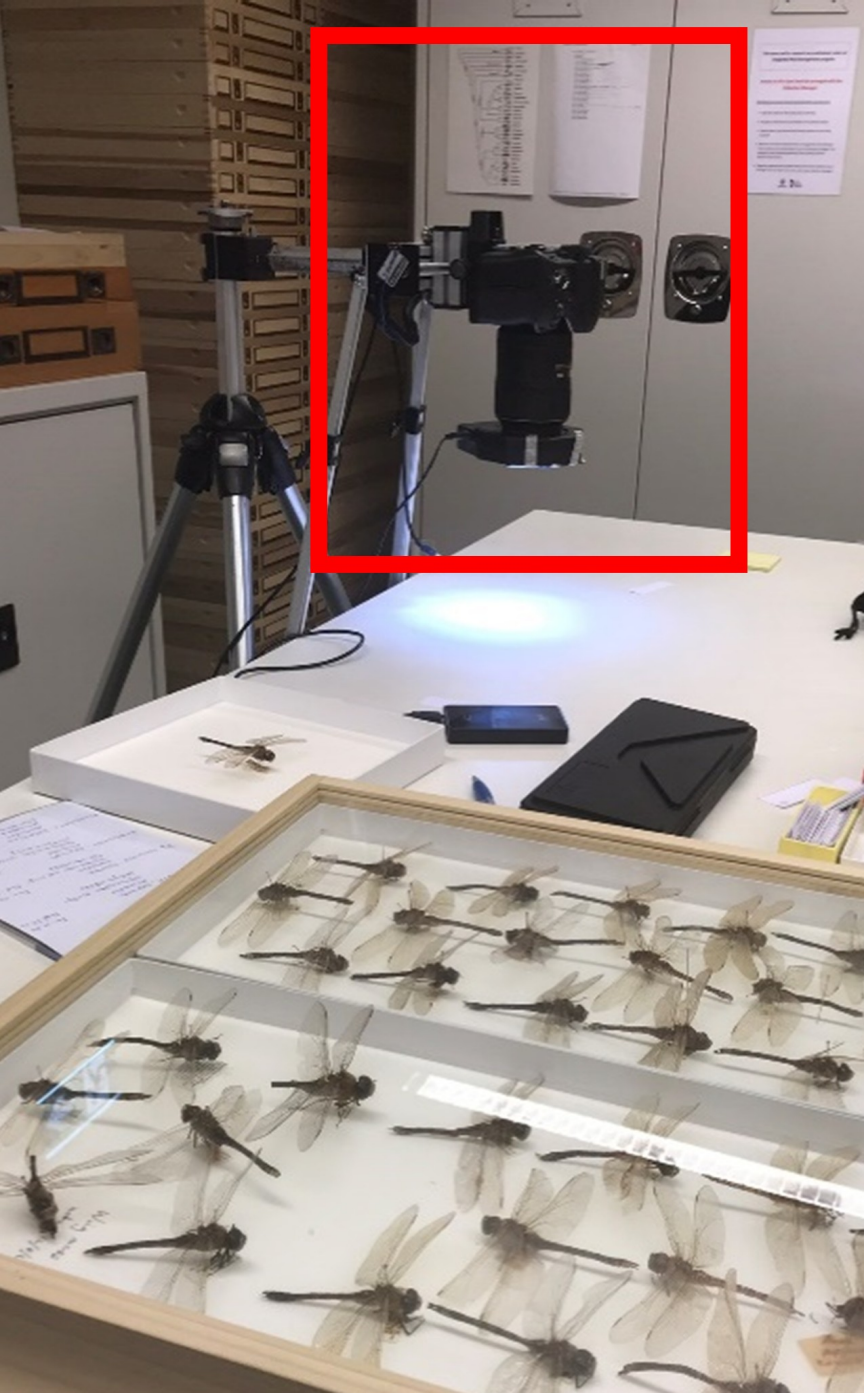
Image capture situation.

The small aperture of f/10 allowed large depth of field images. The overlap of each photograph started from 70% to achieve an accurate 3D model. The distance from the camera to the object was set to 22 cm, and the lens was located above the object with an angle of close to 0° with respect to the vertical. The camera was installed on the linear motion stage that was mounted rigidly on a tripod. The automatic sampling arrangement moved with 1mm steps and at each step sequenced through five different focal lengths for each photograph without manual interaction with the camera or slide to allow a fast, consistent, and accurate result (Fig 4).

A total of 100 individual Odonata from the collection consisting of both suborders, *Zygoptera* and *Epiprocta*, were photographed. The specimens were archived in wooden boxes with glass lids, and the background was a white mat upon which the sample was mounted. The tripod was positioned directly above the dragonfly sample box. The glass lid was found to reflect light sources behind the camera, which affected the results. To solve this issue, a light ring around the lens was used to minimized reflections off the glass. The same light source and approximately the same distance were used for all photographs. From each insect, approximately 100 high-resolution photographs of 4016×6016 pixel resolution from a sequence of positions were automatically captured. Every photograph had the same type of matt background, and the specimens were undisturbed.

### Reconstruction process

The sequence of photographs was used to create a 3D model of the wings. Depending on size, the process took up to 10 minutes per wing to compute a solid model. The procedure was carried out on a high-performance desktop PC with an Intel Corei7 CPU 3.60 GHz, installed memory of 64.0 GB, and also a Graphical Processing Unit or GPU (GeForce GTX 1080 Ti). A variety of photogrammetry software and their available features were evaluated including ARC3D [33], Recap [34], Autodesk 123D [35], MeshLab [36], Visual SFM [37] and 3DF Zephyr [38]. 3DF Zephyr was chosen because it produced a dense point cloud from supplied overlapping images, which provided more detail for the geometry of the wings. Zephyr is a professional reconstruction software that makes use of the GPU with a powerful editing feature. Speed was an important aspect in this study due to the number of Odonata wing specimens. Comparing Zephyr with other similar software options, it was found to support export diverse file formats such as .stl, .ply and .obj. The price was another parameter for choosing this software. The Zephyr was cheaper compared to other paid software and had enough capability available in its demonstration mode to test before purchase, with up to 50 photographs being processed in the free version. By comparison, Visual SFM is a free reconstruction software. However, this software is suitable for aerial images and to obtain detailed texture, secondary software was required and it only had .ply export format. The Autodesk software was another option, but it was more expensive than Zephyr presented photo number limitation. Autodesk also does not have .ply and .stl export format and is a web-based application, which leads to internet dependency, vulnerability and unsuitability for use in remote locations.

**Fig 5:**
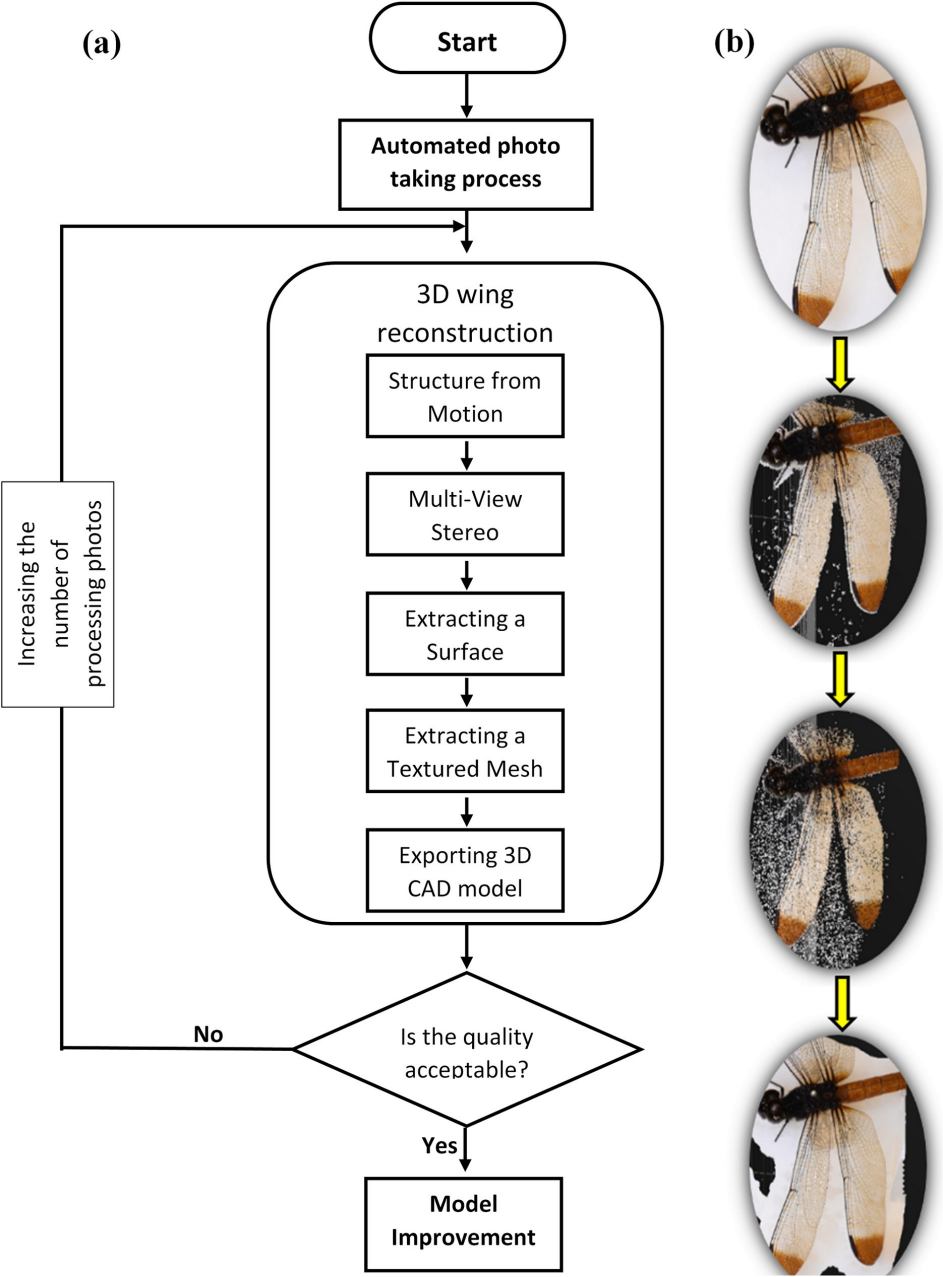
(a) 3D reconstruction of a 2D model of dragonfly wing progress flowchart, (b) 3D reconstruction of a 2D model of dragonfly wing created in Zephyr Pro software taking a photograph, dense point cloud, mesh surface and textured mesh from left to right, respectively.

In 3DF Zephyr, similarly to the laser scanner, the results are provided in a grid in the form of an unordered set of points. Automatic reconstruction of 3D models can also be facilitated by 3DF Zephyr [38]. The process began with putting photos in sequential order for each pair of wings, followed by uploading into the software (Fig 5(B)). Then the dense point cloud, a surface, and textured mesh were extracted in sequence (Fig 5(A)). The calibration of the camera was automatically carried out during analysis by the Zephyr software, using the online pre-calibration function [39]. Zephyr has an integrated interface to the open-source software camera calibration tool 3DF Lapyx [40] from which the intrinsic parameters of a camera are automatically computed [39, 41]. Different 3DF Zephyr settings such as ‟presets”, ‟advanced” and ‟custom” were tested for each step of 3D wing reconstruction during the analysis. Subsequently, according to the results, the ‟advanced” setting was selected due to the close range and the structure of the wing. The first phase was related to the position and orientation of each photo from 100 photos per wing uploaded into Zephyr. The advanced mode was selected using the following settings: “very high keypoint density”, “accurate matching type”, “full matching stage depth”, “incremental reconstruction engine” and “sequential photo ordering”. The next phase was dense point cloud extraction with the refined output type and the number of nearest cameras set as 5. The resolution and noise filtering were 100% and enabled hyperplane matching was considered for advance setting. The following advance settings were used for the last phase: “mesh creation”, “huge polygon count”, “100% of smoothness” and “watertightness and smooth reconstruction type”.

By using the acquired 3D graphics model, it was possible to export the model to several formats such as .stl, .ply, .obj/mtl, etc. These three solid model formats are freely available in the dataset at Dryad (https://doi.org/10.5061/dryad.6t1g1jwtt) repository as a scientific resource.

### Data acquisition

For the presented method, accuracy of reconstruction is the objective. Therefore, the first step was to investigate the accuracy models of the known objects with simpler structure. For this purpose, several objects with known dimensions, including a 1.20mm sized screw pitch gauge, measured using a microscope reticle (± 2%). A block of metal with dimensions 38.10 × 25.05mm (± 0.1%)measured with digital calipers. Good agreement was observed between photogrammetry and direct measurements with a difference of ± 0.03 mm shown in Table 2. The accuracy of the photogrammetric model was 0.1%, as measured from the selected reference standards, which should be acceptable accuracy for many purposes across Odonata wings with a span of 24 to 85 mm.

**Table 2:** Accuracy of photogrammetry on the metal block and screw pitch gauge selected standards.

Created by Zephyr software	Dimension	Dimension by method	Difference	Accuracy
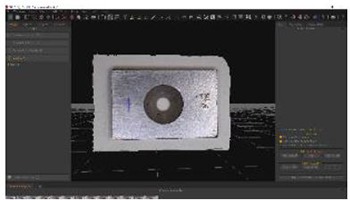	38.10 ×25.05 mm^2^	38.09 ×25.02 mm^2^	±0.01×0.03 mm^2^	99.9%
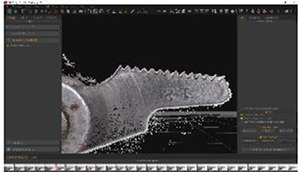	1.2 mm pitch dimension	1.17 mm pitch dimension	±0.03 mm	98%

A useful reference standard for mechanical engineering purposes is a flat surface. Flatness is a defined property from which deviations can be measured. To test the performance of the photogrammetric technique against a structure with similar size and venation pattern, an ornament with a flat transparent plastic veined wing was digitized using the method. The wing was clamped using a vacuum table to prevent local warping.

### Micro-CT

The precision of the presented method in surface dimensions was shown to be satisfactory in Table 2; however, the accuracy of the depth and angles of the corrugation pattern needed to be evaluated to ensure that the model is accurate. For this purpose, micro-computed tomography (Micro-CT) scanning was used. The hindwing of *Orthetrum caledonicum* from the *Libellulidae* family was carefully excised from the body with a razor blade. The iodine staining method was used to visualize the transparent wing by x-ray micro-tomography overnight and then dried at room temperature for another 24 hours. The Xradia MicroXCT 400 Micro Tomography system (ANFF-SA Node, University of South Australia, Australia) was employed, and the wing was scanned at 16μm resolution with a 40-kV voltage source. The scanning process took 18 hours per wing. The exported data from the scanner were analyzed and edited by Avizo image processing software. In addition, two more specimens *Cordulegaster Boltonii* from *Cordulegastridae* family and *Neurobasis Chinensis Indonesia* from *Calopterygidae* were collected and Micro-CT was conducted on them.

## Results and discussion

The 3D point clouds obtained from photogrammetry were very dense due to a large number of image features and could be discerned relatively quickly, depending on the computational power of the computer and software [42]. Our results showed that 3D Zephyr can reliably fuse images of parts of an object into a complete 3D model [43]. Tests were undertaken to establish an appropriate technique before deciding that photogrammetry was necessary for the sampling scenario of collections [44].

**Fig 6:**
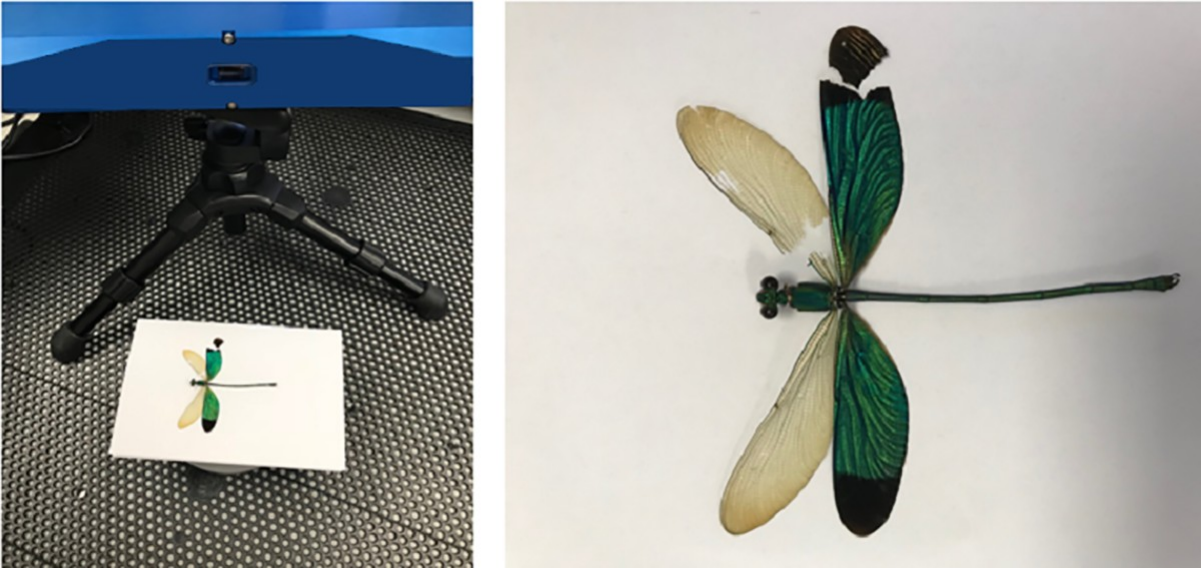
Three-dimensional laser scanning of the right side of the *Neurobasis daviesi* wing.

A *Neurobasis daviesi* was caught and analyzed with two different 3D measurement methods, laser range finding and photogrammetry. After taking photographs with a camera, the LMI Technologies HDI 120 3D laser scanner was also applied to a specimen. Considering the dragonfly wing is semi-transparent, 3D reconstruction using laser ranging based on triangulation of points is unreliable [45]. Other issues regarding 3D laser scanning include unreliability against a white background and the need to pigment the wing resulting in destruction of the sample [46]. Finally, the 3D scanner is an expensive apparatus and method [35]. The results indicate that 3D laser scanning might be a proper method for digitizing large objects such as the human body, or robust objects such as wood or stone, but not delicate insect wings [47]. As shown in Fig 6, the laser scanner destroyed the wing sample and cut it when the device was turned on.

**Fig 7:**
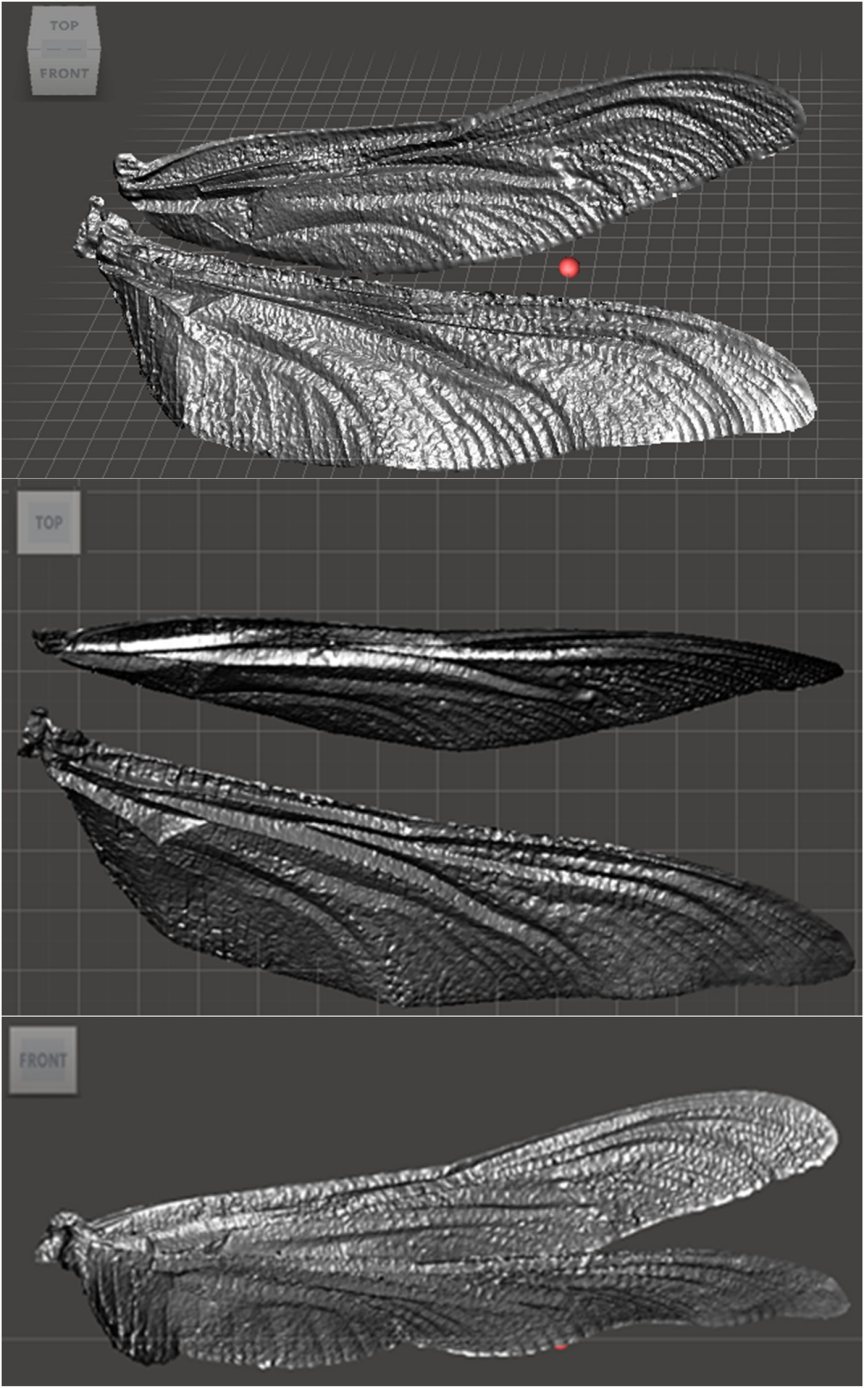
3D geometry of the isolated fore and hind wing of *Petaluridae*, *Petalura*, the dimension of fore wing and hind wing are 84.42 and 84.77 respectively.

Fig 7 shows the final photogrammetric 3D reconstruction of wings of a *Petaluridae Petalura*. The method shows a consistent shape but there remained a need to validate the technique against targets of approximately the same size and optical properties.

**Fig 8:**
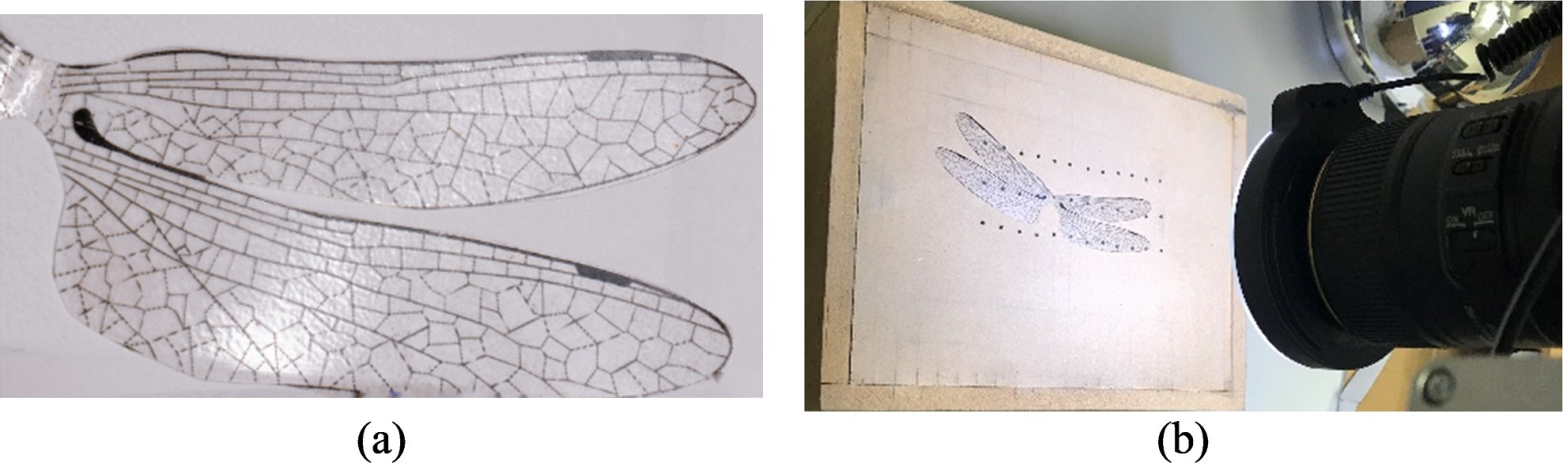
(a) Transparent flat wing and (b) vacuum table setup.

To calibrate the method, a transparent flat wing from a toy with a span length of 54 mm (Fig 8(A)) was used as an experimental control to minimize the height variations and to obtain the accuracy of the proposed method. For a more accurate result, a vacuum table was used for holding the plastic wing without any movement (Fig 8(B)).

### Corrugation pattern validation

**Fig 9:**
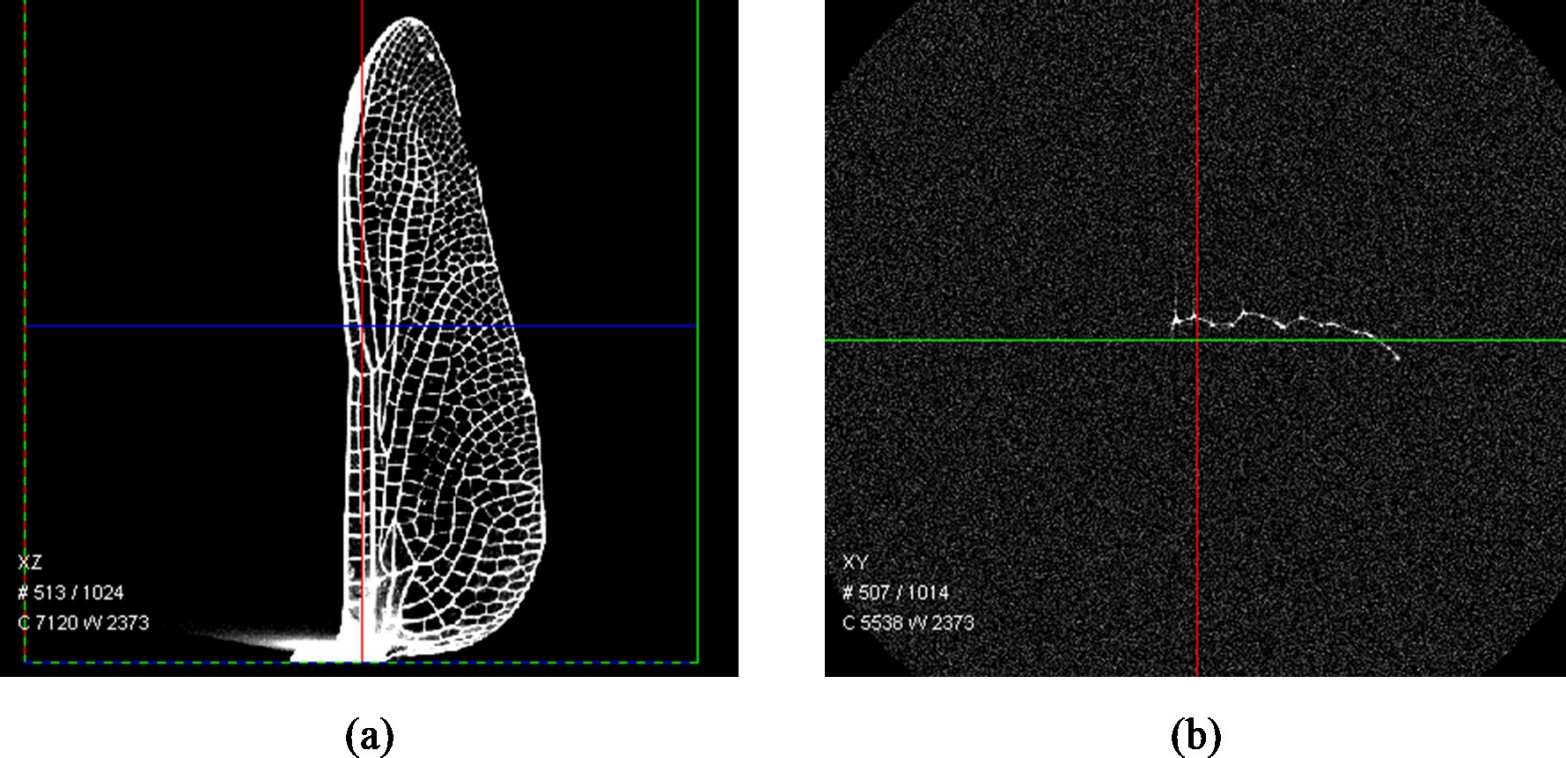
(a) 3D visualization and (b) cross-section of the HW of the *Orthetrum caledonicum* obtained by Micro-CT.

Fig 9 shows the visualization of the hind wing of an *Orthetrum caledonicum*, which was scanned by Micro-CT and reconstructed.

**Fig 10:**
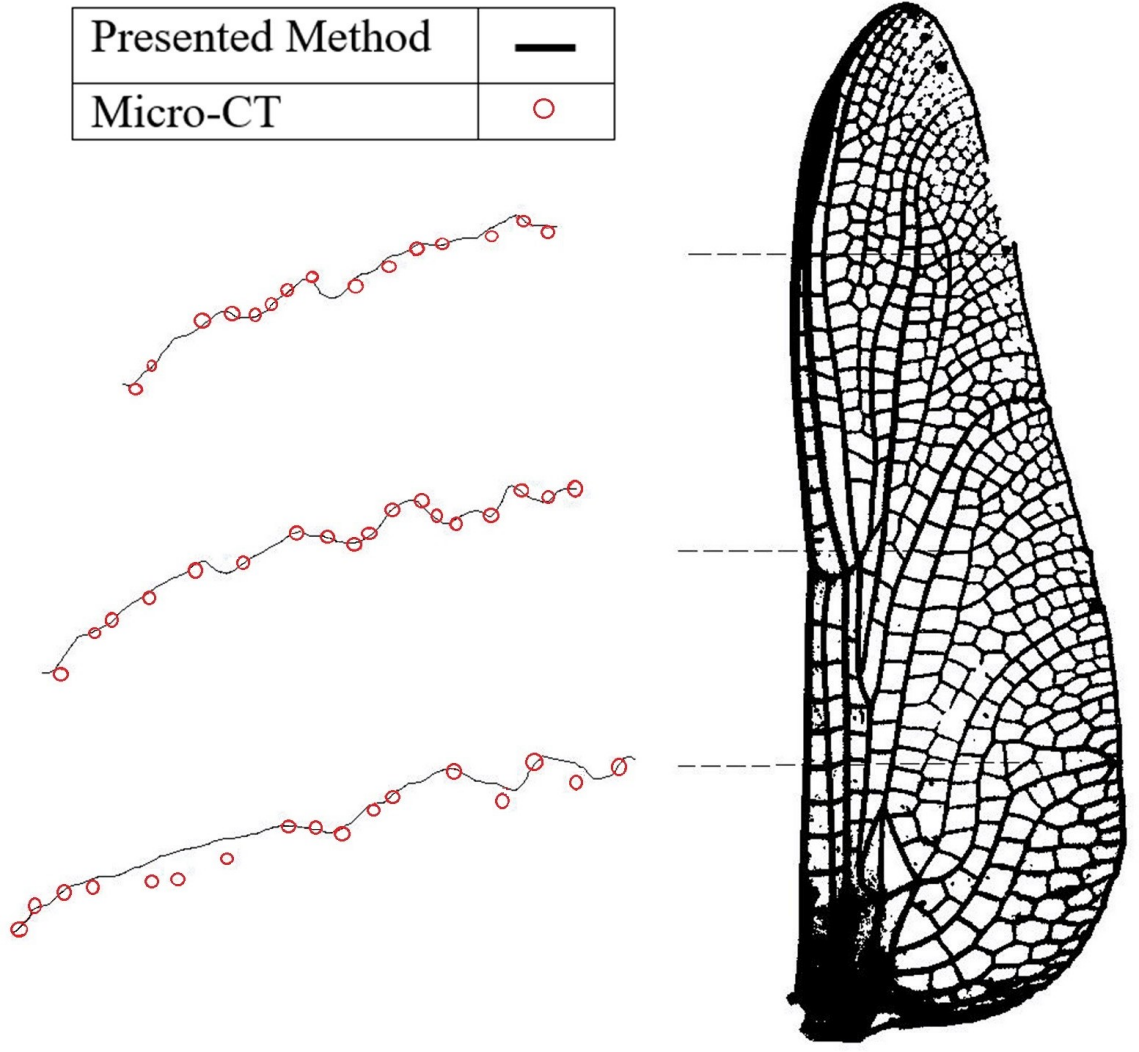
Corrugation structures measured at different sections along the HW of the *Orthetrum caledonicum* obtained by photogrammetry and Micro-CT.

The 3D model of the same wing created using the photogrammetric method was reconstructed and wing sections from different span-wise stations were compared with the results obtained by Micro-CT analysis, as shown in Fig 10. It is evident that the corrugation profiles obtained by the presented method are well-matched to those from the Micro-CT. There are some discrepancies in the areas of the wing that are not heavily corrugated. We suggest that these discrepancies are due to an absence of stabilizing ridged structure allowing pose of the wing, staining treatment, humidity, and dehydration over time to change the shape of the wing. The photogrammetric technique was completed within minutes of the wing excision, the Micro-CT scan was completed days afterward on the same sample.

**Fig 11:**
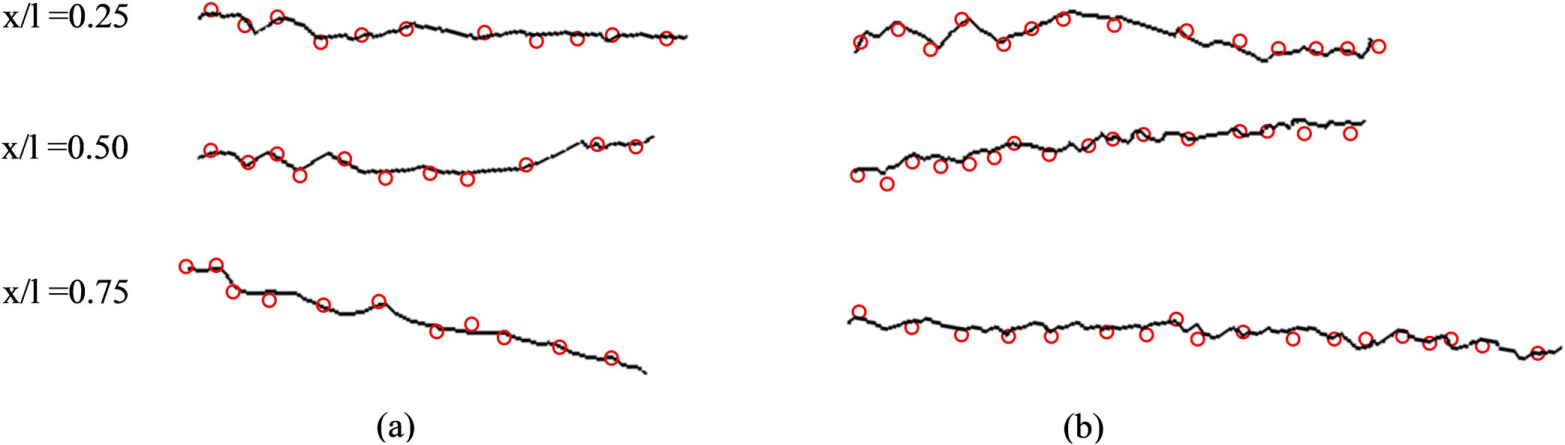
Corrugation patterns at three stations along the HW of (a) *Cordulegaster Boltonii* and (b) *Neurobasis Chinensis Indonesia* obtained by photogrammetry and Micro-CT.

Fig 11 shows the corrugation patterns of different sections of two more specimens, including (a) *Cordulegaster Boltonii* and (b) *Neurobasis Chinensis Indonesia*, are well-matched.

In order to quantify the difference between the corrugation patterns obtained from both photogrammetry and micro-CT methods, the Sum of Squared Difference (SSD) was calculated for wing sections, which are shown in Fig 10 and Fig 11. For this purpose, the sections were digitized, and the coordinates (x, y) of the points on the airfoils for both methods were extracted. Also, the distance between one measured position and the next was set to 1 mm. Then, SSD was calculated for each section individually. The vertical coordinates of the points extracted from the results of both methods at the same amount of x were non-dimensionalized to the length of the section.

The average amount of SSD for the studied wing sections was around 0.0041 mm, with a standard deviation of 0.0014 mm, which seems to be acceptable for most purposes. Note that, the root-section of the wing from the *Orthetrum caledonicum* species was excluded from the calculation. The reason for that is the calculated SSD for this section was far from the rest of the sections and seems to cause an unrealistic average SSD for the presented method. However, even including the mentioned sections results in an SSD equal to 0.005 mm, which is still a low error.

By comparing the Micro-CT and photogrammetry methods, it was found that Micro-CT is a time consuming and expensive process for 3D reconstruction and still involves image processing data analysis and cleaning the mesh and surface.

### Corrugated wing fabrication

Photogrammetrically captured corrugation patterns of the wings appeared to have acceptable precision for engineering simulation. Manufacturing of the corrugated wing from the 3D CAD model is potentially useful for building small aircraft.

We were interested to assess the ability of current manufacturing methods to build wings from 3D model files. To that end, two different methods were examined, direct printing on a 3D printer and making of an aluminum billet die using a CNC mill. The fabricated wings were suitable for experimental flow visualization.

**Fig 12:**
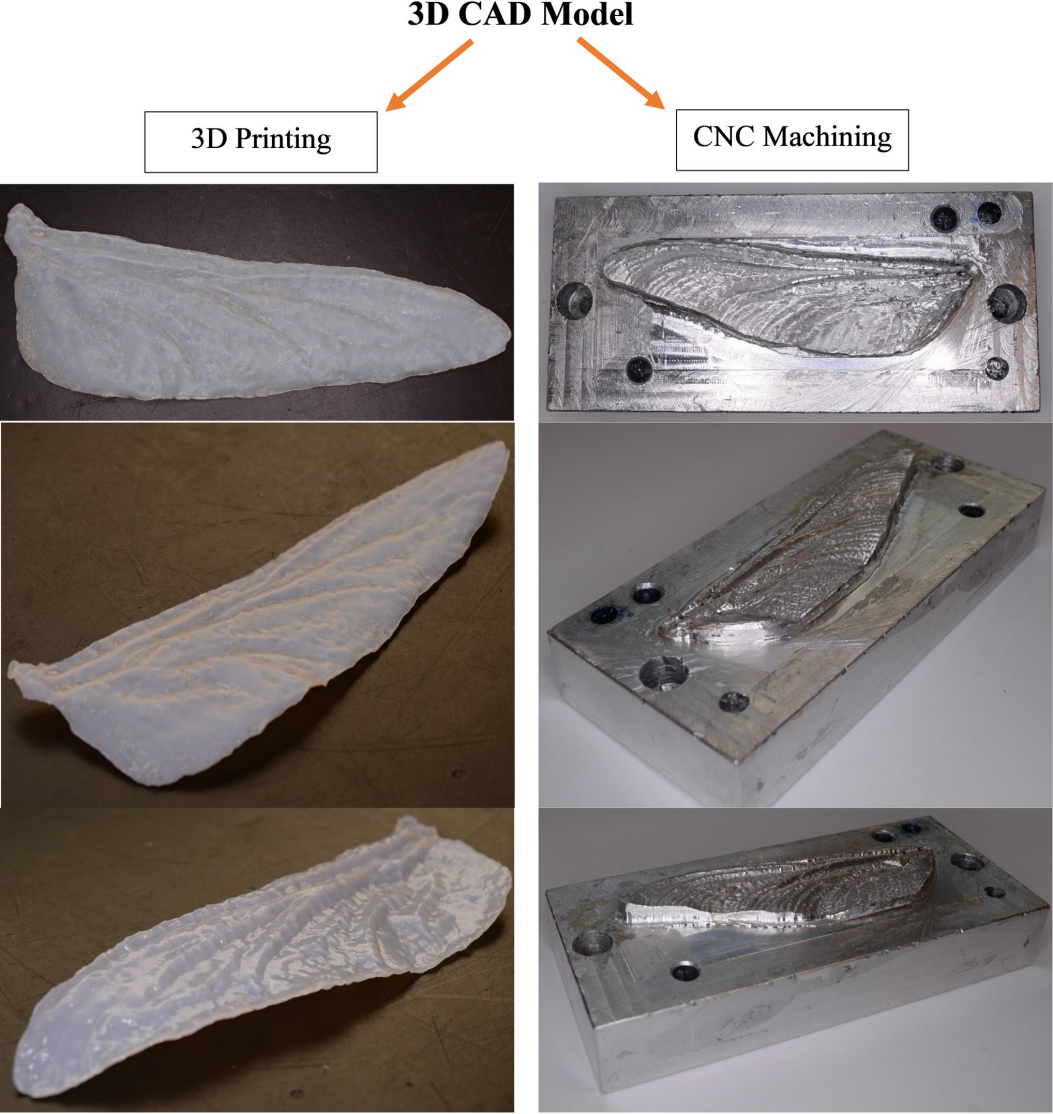
Forewing dragonfly 3D printed and Forewing dragonfly mold made by CNC.

The 3D printing time for one wing on a 3D printer (S1 File) was much less than for milling the die with the CNC machine (Fig 12). The CNC die, once created, allowed repeatable production of wings from various materials at a high rate.

**Fig 13:**
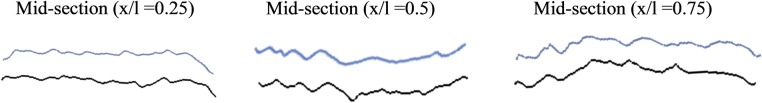
Comparison of wings at three section of the 3D model vs fabricated wing.

The rapid prototype Stereolithography Apparatus (SLA) printer, Stratasys Objet30 V3 model, was used at the University of South Australia to manufacture the flexible 3D model of the bioinspired wings. VeroGray material was selected to fabricate the wing due to lightweight, transparency, strength and flexure, high accuracy and durability. After the wing model was fabricated by 3D printer, the photogrammetry technique was applied to the fabricated wing to evaluate the manufacturing process and the accuracy of the fabricated model. Fig 13 shows the corrugation patterns of the 3D model (blue line) and fabricated wing (black line) at three sections; 25%, 50% and 75% of the wingspan from the body. The isometric view of the same model is presented in Fig 12. The same process was applied to calculate the difference between 3D geometry and the 3D printed model. The SSD and standard deviations are 0.009 mm and 0.001 mm (Fig 13). It is found that the corrugations are still well matched specifically in main corrugations but there are some defects due to fabrication limitations and possibly warping of the part.

According to measurements of the angle and dimension of the screw pitch gauge, it is evident that the trend and angle of the corrugation of the wings are preserved after digitization, further confirming the accuracy of the database we have created. Also, the measurements and model generation appear to be accurate enough to use for aeronautical analysis of flight performance and maneuverability. The underside of the wing could not be captured due to limitations of access to samples in the collection. However, considering the digitized geometry and thinness of the dragonfly wing, any difference related to that is probably negligible or subject to being characterized. Future work needs to focus on the analysis of the meshing, modeling, and inferring hidden details on the wing. Also, the ability to mimic the real dragonfly including all the features and venation along the wing are topics for future investigations.

## Conclusion

This study created a unique dataset including detailed 3D geometry from museum specimens of Odonata wings with high repeatability and accuracy. The current database represents the application of the non-destructive methodology to 80 species of Odonata and is the result of processing over 8000 images with extensive post-processing. The accuracy of the presented method was evaluated against Micro-CT, showing agreement between the results. The 3D models were used to produce a 3D printed wing, and CNC machined die for the fabrication of wings. This low-cost, non-contact method for close-range photogrammetry was proven to be effective in the measurement of 3D objects in difficult circumstances inside cases and behind glass.

Both the dataset and technique are of value for ecological and evolutionary studies of complex functional structures, but also for a wide range of engineering studies such as bioinspired aircraft design and computational studies of structural response to airflow.

## Supporting information

S1 Table Full description of Odonata samples used in the study. (DOCX)

S1 File The .stl file of 3D printed wing. (STL)
