# Supplementary material for: Experimental method for 3D reconstruction of Odonata wings (methodology and dataset)
Source: PLoS One. 2020 Apr 29;15(4):e0232193. doi: 10.1371/journal.pone.0232193 (PMC7190169; doi:10.1371/journal.pone.0232193)
Supplement: S1 Table — (DOCX) [file pone.0232193.s001.docx]

**S1 Table. Full description of Odonata samples used in the study**

| Order | Suborder | *Family* | *Species* |
| --- | --- | --- | --- |
| Odonata | Epiprocta | Aeshnidae | Adversaechna (Rambur, 1842) |
| Odonata | Epiprocta | Aeshnidae | Anax Guttatus (Burmeistwer, 1839) |
| Odonata | Epiprocta | Aeshnidae | Austroaeschna Multipunctata (Martin, 1901) |
| Odonata | Epiprocta | Aeshnidae | Austroaeschna Parvistigma (Selys, 1883) |
| Odonata | Epiprocta | Aeshnidae | Gynacantha Kirbyi (Kruger, 1898) |
| Odonata | Epiprocta | Aeshnidae | Gynacantha Mocsaryi (Froster, 1898) |
| Odonata | Epiprocta | Aeshnidae | Dendroaeschna Conspersa (Tillyard, 1907) |
| Odonata | Epiprocta | Aeshnidae | Hemianax (Burmeister, 1839) |
| Odonata | Epiprocta | Aeshnidae | Notoaeschna Sagittata (Martin, 1901) |
| Odonata | Epiprocta | Aeshnidae | Telephlebia Godeffroyi (Selys, 1883) |
| Odonata | Epiprocta | Aeshnidae | Accessions |
| Odonata | Epiprocta | Cordulegastridae | Cordulegaster Boltonii (Donovan, 1807) |
| Odonata | Epiprocta | Corduliidae | Epicordulia Princeps (Nagen, 1861) |
| Odonata | Epiprocta | Corduliidae | Hemicordulia Australiae (Rambur, 1842) |
| Odonata | Epiprocta | Corduliidae | Hemicordulia (Selys, 1971) |
| Odonata | Zygoptera | Accessions-Foreign | Japan & Formosa |
| Odonata | Zygoptera | Accessions-Foreign | Japan & Formosa |
| Odonata | Zygoptera | Accessions-Foreign |  |
| Odonata | Zygoptera | Protoneuridae | Protoneuridae |
| Odonata | Zygoptera | Megapodagriondae | Podopteryx Selysi (Froster, 1899) |
| Odonata | Zygoptera | Lestidae | Diphlebia |
| Odonata | Zygoptera | Lestidae | Diphlebia Nymphoidaes (Tillyard, 1912) |
| Odonata | Zygoptera | Calopterygidae | Neurobasis Australis (Selys, 1897) |
| Odonata | Zygoptera | Calopterygidae | Neurobasis Chinensis Indonesia (east Java) |
| Odonata | Zygoptera | Calopterygidae | Calopteryx Maculata (Palisot de Beauvois, 1805) |
| Odonata | Zygoptera | Calopterygidae | Calopteryx Haemorrhoidalis |
| Odonata | Zygoptera | Chlorocyphidae | Playcypha |
| Odonata | Zygoptera | Coenagrionidae | Papuagrion Occipitale (Selys, 1877) |
| Odonata | Epiprocta | Libellulidae | Libellula Pulchella (Drury, 1773) |
| Odonata | Epiprocta | Libellulidae | Libellula Indica (Fabricius, 1781) |
| Odonata | Epiprocta | Libellulidae | Libellula Saturata (Uhler,1857) |
| Odonata | Epiprocta | Libellulidae | Neurothemis Stig Matizans Bramina (Guerin-meneville,1832) |
| Odonata | Epiprocta | Libellulidae | Neurothemis Oligoneura (Brauer, 1867) |
| Odonata | Epiprocta | Libellulidae | Orthetrum Caledonicum (Brauer, 1865) |
| Odonata | Epiprocta | Libellulidae | Orthetrum Glaucum (Brauer, 1865) |
| Odonata | Epiprocta | Libellulidae | Orthetrum Villosovittatum (Brauer, 1868) |
| Odonata | Epiprocta | Libellulidae | Pantala Flave Scens (Fabricius,1798) |
| Odonata | Epiprocta | Libellulidae | Lathrecista Asiatica (Fabricius,1798) |
| Odonata | Epiprocta | Libellulidae | Protorthemis Coronata (Kaup in brauer,1866) |
| Odonata | Epiprocta | Libellulidae | Plathemis Lydia (Drury,1773) |
| Odonata | Epiprocta | Libellulidae | Rhodothernis (Lieftincki fraser,1954) |
| Odonata | Epiprocta | Libellulidae | Phyothemis Graphipters |
| Odonata | Epiprocta | Libellulidae | Phyothemis Phyllis |
| Odonata | Epiprocta | Libellulidae | Phyothemis Princeps |
| Odonata | Epiprocta | Libellulidae | Tholymis Tillarga |
| Odonata | Epiprocta | Libellulidae | Tramea Loewii |
| Odonata | Epiprocta | Libellulidae | Zyxomma Sp |
| Odonata | Epiprocta | Libellulidae | Hydrobasileus Brevistylus |
| Odonata | Epiprocta | Libellulidae | Orthetrum caledonicum |
| Odonata | Epiprocta | Libellulidae | Crocothemis Nigrifrons |
| Odonata | Epiprocta | Libellulidae | Diplacina Phoebe |
| Odonata | Epiprocta | Libellulidae | Camacinia Othello |
| Odonata | Epiprocta | Libellulidae | Agrionopters Longitudinalis |
| Odonata | Epiprocta | Libellulidae | Agrionoptera Insignis |
| Odonata | Epiprocta | Libellulidae | Celithemis Eponina |
| Odonata | Epiprocta | Gomphidae | Accessions |
| Odonata | Epiprocta | Gomphidae | Ictinogomphus Australis |
| Odonata | Epiprocta | Gomphidae | Hemigomphus Gouldii |
| Odonata | Epiprocta | Gomphidae | Gomphus Crassus |
| Odonata | Epiprocta | Gomphidae | Dromogomphus Spoliatus |
| Odonata | Epiprocta | Gomphidae | Austrogomphus Prasinus |
| Odonata | Epiprocta | Corduliidae | Procordulia |
| Odonata | Epiprocta | Corduliidae | Procordulia Jacksoniensis |
| Odonata | Epiprocta | Corduliidae | Procordulia |
| Odonata | Epiprocta | Macromiidae | Macromia Tillyardi |
| Odonata | Epiprocta | Macromiidae | Macromia Illinoiensis |
| Odonata | Epiprocta | Macromiidae | Macromia Taeniolata |
| Odonata | Epiprocta | Petaluridae | Petalura Ingentissima |
| Odonata | Epiprocta | Petaluridae | Petalura Gigantea |
| Odonata | Epiprocta | Petaluridae | Uropetala Carovei |
| Odonata | Epiprocta | Synthemistidae | Eusynthemis Brevistyla |
| Odonata | Epiprocta | Synthemistidae | Choristhemis Flavoterminate |
| Odonata | Epiprocta | Synthemistidae | Archaeosynthemis Orientalis |
| Odonata | Epiprocta | Epiprocta | Accessions-New Guinea |
| Odonata | Epiprocta | Epiprocta | Accessions-New Guinea |
| Odonata | Epiprocta | Epiprocta | Accessions-New Guinea |
| Odonata | Epiprocta | Accessions |  |
| Odonata | Epiprocta | Accessions |  |
| Odonata | Epiprocta | (Accessions) |  |
| Odonata | Epiprocta | (Accessions) |  |
